# Supplementary material for: Detection of Salmonella Enterica in Egg Yolk by PCR on a Microfluidic Disc Device Using Immunomagnetic Beads
Source: Sensors (Basel). 2020 Feb 15;20(4):1060. doi: 10.3390/s20041060 (PMC7070913; doi:10.3390/s20041060)
Supplement: Supplementary file 1 [file sensors-20-01060-s001.pdf]

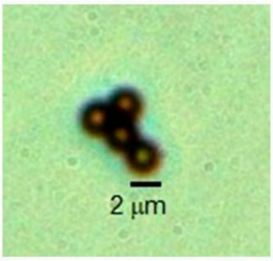

Supplement Fig. 1 Optical microscopic image of immune magnetic beads
The image was acquired through a CCD camera (Axio cam MRc, Carl Zeis) and imaging system Axio vision 4.8). The size was measured using the imaging system.
